# Humans Dominate the Social Interaction Networks of Urban Free-Ranging Dogs in India

**DOI:** 10.3389/fpsyg.2020.02153

**Published:** 2020-08-25

**Authors:** Debottam Bhattacharjee, Anindita Bhadra

**Affiliations:** Department of Biological Sciences, Indian Institute of Science Education and Research Kolkata, Kolkata, India

**Keywords:** human-animal interaction, dog–human interaction, free-ranging dogs, human flux, social network analysis

## Abstract

Research on human-animal interaction has skyrocketed in the last decade. Rapid urbanization has led scientists to investigate its impact on several species living in the vicinity of humans. Domesticated dogs (*Canis lupus familiaris*) are one such species that interact with humans and are also called man’s best friend. However, when it comes to the free-ranging population of dogs, interactions become quite complicated. Unfortunately, studies regarding free-ranging dog–human interactions are limited even though the majority of the world’s dog population is free-ranging. In this study, we observed twelve groups of free-ranging dogs in their natural habitat, the streets. We quantified their interactions at the intra (dog–dog) and interspecific (dog–human) levels. The study areas were divided into two zones, namely – intermediate and high flux, based on human activity or movement. Social network analysis revealed higher instances of interspecific than intraspecific interactions, irrespective of the human flux zones. Humans, in significantly higher occasions, initiated both positive and negative behaviors in comparison to dogs. Our findings conclude that humans are a crucial part of the interaction network of Indian free-ranging dogs.

## Introduction

Domesticated dogs (*Canis lupus familiaris*) have a long and rather intricate co-evolutionary relationship with humans (Vilà et al., 1997; Savolainen et al., 2002; Thalmann et al., 2013; Frantz et al., 2016). Dogs and humans share very warm social relationships, driven primarily by the abilities of dogs to communicate with humans, much more than any other species. Domestication has been proposed to be a critical factor in facilitating dogs’ ability to read human cues and gestures (Hare et al., 2002; Hare and Tomasello, 2005). Numerous studies have also pointed out the role of ontogeny, through shared experiences with humans, in developing such capabilities in dogs (Wynne et al., 2008; Udell, 2015). Consequently, researchers have concluded that such high degree of socio-cognitive skills is probably the result of the dual influence of evolutionary history and ontogenic experience of individuals through socialization (Gácsi et al., 2009; Lampe et al., 2017). More recent studies have postulated that these skills of dogs could also be attributed to their “differential behavior” (Range et al., 2019) leading to increased conflict-avoidance and “hypersociability” (VonHoldt et al., 2017) accounting for their greater sociability toward humans. Undoubtedly, these socio-cognitive skills have enabled dogs’ successful co-existence with humans.

Despite significant advancement in the understanding of the evolution of the dog–human relationship, little is understood of how the majority of the world’s dog population interact with humans. Nearly 80% of the world’s dog population is represented by free-ranging dogs, found in the global south (Hughes and Macdonald, 2013; Lord et al., 2013). They occur as natural breeding populations living without direct human supervision (Serpell, 1996; Bonanni et al., 2011; Bonanni and Cafazzo, 2014). Free-ranging dogs are also genetically more diverse and geographically widespread than purebred dogs (Shannon et al., 2015). Free-ranging dogs are scavengers, depending primarily on human-generated resources for subsistence (MacDonald and Carr, 2016). Unlike other urban-adapted animals that maintain a wary distance from humans (Rodewald and Shustack, 2008; Carrete and Tella, 2011; Samia et al., 2017), free-ranging dogs have been shown to interact with humans regularly. Therefore, exploring the various facets of free-ranging dog–human interactions can help us understand the evolution of the dog–human relationship better.

In India, free-ranging dogs have been living for many centuries as a natural population (Debroy, 2008; Shannon et al., 2015). They are present in every possible human habitat, from forest fringes to metropolitan cities (Vanak and Gompper, 2009; Gompper, 2013; Bhattacharjee et al., 2020a). The relationship of these dogs with humans is quite complex and multidirectional, ranging from very negative to very positive. For example, free-ranging dogs are potential reservoirs of various zoonotic diseases, including rabies, posing a threat to humans and the wildlife (Belsare et al., 2014; Gompper, 2015; Home et al., 2017). Moreover, they scatter garbage, defecate in open spaces, and bark at night, thus being considered as a menace. Humans, on the other hand, influence the behavioral dynamics of free-ranging dogs too. These dogs are often beaten, threatened and even killed by humans (Paul et al., 2016). Still, they choose dens close to human habitats (Sen Majumder et al., 2016) and are cared for by some humans as well. Also, there are several groups across the country, mostly in large cities, working toward the welfare of free-ranging dogs (Totton et al., 2010; Demirbas et al., 2017). These dogs not only scavenge among refuse but use active begging from humans as a strategy for foraging (Bhadra et al., 2016). A recent pan-India survey revealed a significant variation in the human perception of free-ranging dogs across different human habitats (Bhattacharjee et al., 2020a). Hence, investigating the direct interactions between free-ranging dogs and humans can provide us with significant mitigation measures on the conflict of the two species.

Though free-ranging dogs are not owned and do not undergo training or habituation to particular humans, the urban habitats provide an environment for varied interactions between humans and free-ranging dogs in India. A series of studies have investigated the socio-cognitive skills of free-ranging dogs, emphasizing their relationship with humans. For example, their ability to follow simple and complex human pointing gestures (Bhattacharjee et al., 2017a, 2019). Additionally, they have been shown to display situation-specific responsiveness to typically used human social cues (Bhattacharjee et al., 2018, 2020b). In urban habitats, free-ranging dogs regularly encounter unfamiliar humans and experience a range of behaviors. A study concluded that these dogs do not establish physical contact with unknown humans in the first place, but, trust-building can happen with repetitive social contact within a short span of time (Bhattacharjee et al., 2017b). In a recent study, we found that the sociability of these dogs is correlated with human flux or movement in a given area (Bhattacharjee et al., 2020a). We concluded that dogs in the intermediate human flux zones, typically represented by urban neighborhoods, are more sociable in comparison to dogs in high human flux zones, represented by areas like railway and bus stations, marketplaces, etc. In the intermediate human flux zones, sociability, thus could probably be a response to higher positive dog–human interaction than the other zones. It is also necessary to understand that we did not quantify the negative interactions and did not have information on the ontogenic experience of the dogs. Therefore, the underlying reasons for such variation in sociability were not assessed; we assume that direct interactions between dogs and humans would be the first step to have some valuable insights.

In this study, we carried out behavioral observations on groups of free-ranging dogs in intermediate and high human flux zones. We recorded their activities in terms of intra-(dog–dog) and interspecific (dog–human) interactions and subjected these to social network analysis (SNA). SNA is a powerful tool which can be used to understand various patterns of interactions among social animals (Wasserman and Faust, 1994). Given their complete dependence on humans for sustenance, we hypothesized that humans are a crucial part of the interaction network of free-ranging dogs in urban environments. As higher flux of humans may result in higher interactions with dogs, we expected to observe a higher frequency of interspecific interactions (both positive and negative) in the high flux zones, as compared to the intermediate ones. However, since the dogs are typically not very active (Sen Majumder et al., 2014b), the data obtained was not large enough to predict the detailed dynamics of intra and interspecific interactions.

## Materials and Methods

### Study Area and Subjects

We conducted the study in different parts of the following two cities – Bengaluru (12°97′16″N and 77°59′46″E), Karnataka and Raiganj (25°63′29″N, 88°13′19″E), West Bengal, India. We used a “zone categorization criterion” (HF: ≥ 60; 60 < IF ≥ 10) developed earlier by us (Bhattacharjee et al., 2020a) to identify intermediate and high human flux zones. Based on the criterion, we defined high and intermediate flux areas, where human movements were ≥60, and <60 to >10 per minute, respectively. We typically considered crowded areas like market places, bus and railway stations for high, and partial residential areas with shops for intermediate human flux zones. We chose random spots in the areas and stood there for 1 min between 1600 – 1800 h to count the number of people and vehicles that passed by. We repeated the process at least five times to calculate the average human flux in each area. The process was consistent for characterizing the study areas. We randomly selected six dog groups (average group size: 6.5 ± 2.88) in the intermediate and 6 groups (9 ± 4.38) in the high human flux zones (Supplementary Table S1). Groups were defined when dogs were either sitting or moving together within a distance of ≤1 m of each other (Sen Majumder et al., 2014a). All the groups were mixed-sex (male-female) groups and distantly located from each other (Supplementary Figure S1), without any possibilities of interactions. Observations were carried out between June 2018 to August 2019.

### Observations on Dog Groups

We used a mixture of 5-min All Occurrences Sessions (AOS) and 1-min Instantaneous Scan session interspersed by 2-min breaks to carry out focal group sampling of behavior during the study (Altmann, 1974). We recorded the behavioral “events” or interactions between the focal group members and with humans using AOS data. Scan data were obtained to have information on the behavioral “states,” not events. For this study, we only used data from AOS, emphasizing interactions. However, for convenience, we have reported the complete method of sampling here. Each group was observed for 24 observation sessions of 2 h duration each, distributed over different days. Each 2-h session thus had 12 AOS, and 12 scans, distributed randomly and pre-prepared sheets (with randomized AOS and Scan timeslots) were used for recording data to minimize observer bias (Gadagkar, 2001). The observations were carried out in different time slots (0700 – 0900 h, 1000 – 1200 h, 1300 – 1500 h, and 1600 – 1800 h), to cover most of the time when humans and dogs are likely to interact on the streets (Sen Majumder et al., 2014a).

Observations were carried out on each of the different timeslots six times, summing up to a total observational period of 48 h for a focal group. However, we pooled the data from the different timeslots for our analyses. None of the groups was observed more than twice (also not on consecutive timeslots) on a particular day. Since we were interested in understanding dog–human interactions, we did not investigate the effects of different seasons like pre-mating, mating, and pup-emergence (Sen Majumder et al., 2014a), which may influence the intraspecific dynamics of dogs. Moreover, 1-year long observation of groups enabled us to capture general information on dogs’ interspecific interactions with humans. The observation was done from a certain distance (not less than 15 m) in order to avoid influencing the dogs.

### Data Analysis

We noted all the intra (dog–dog) and interspecific (dog–human) behaviors from the AOS and subjected these to SNA and statistical modeling.

(a) Behavior – Intraspecific behaviors were considered when members of a focal dog group interacted among each other. However, differentiation was not done between the types of intraspecific interactions, e.g., agonistic or affiliative. We only counted the number of instances when such interactions occurred. Interspecific behaviors, on the other hand, were defined when members of a focal dog group interacted with humans. Since dog–human interaction was the primary focus, we quantified all possible components of the interactions, including directionality. Dog – human interaction on the streets can be bidirectional, where both humans and dogs can initiate behaviors toward each other. We further subdivided the behaviors into two major categories – positive and negative. We summarized all the interactive behaviors that are typically seen between dogs and humans –

• *Dog – induced positive –* Positive or affiliative behaviors by dogs directed toward humans. Behaviors included any of the following – gazing with tail-wagging, begging while standing or sitting close to humans (≤0.3 m), and licking humans with tail-wagging.
• *Dog – induced negative –* Agonistic behaviors shown toward humans by dogs. It included either of the following behaviors – attacking humans, barking, chasing, snarling, growling, and biting.
• *Human-induced positive –* Affiliative behaviors exhibited toward dogs by humans. It primarily included positive social petting and food provisioning by humans. Positive vocalizations (Bhattacharjee et al., 2017b) from humans were also considered.
• *Human-induced negative –* Negative behaviors showed by humans toward dogs. This included threatening of dogs by various means, beating, and shooing away (Bhattacharjee et al., 2018, 2020b).

(b) Network analysis – SNA was performed to visualize and subsequently analyse the data. Following are the brief details of the network properties used in the analysis –

*Network*–A total of 12 networks were generated in this study. Every individual dog in a focal group was considered as a node. Additionally, we defined “humans” as nodes in all the networks. It should be noted that the node “human” represented the species, and thus did not have an individual identity. Therefore, each network consisted of *n* + 1 nodes (*n* = number of dogs in a focal group, and 1 = an additional node denoting all humans that the individuals in the group were seen to interact with). Edge was defined by a line or link between two nodes, illustrating an interaction.

*Node strength*–Node strength was used to designate the number of edges or weight between two nodes. For example, if node “*i*” interacted with node “*j*” 5 times, it would have a strength of five. We calculated the node strength for intra and interspecific interactions separately. Besides, we measured the strength of the positive and negative behaviors induced by dogs and humans toward each other for the interspecific interactions. In this study, all the edges in the networks were directional and weighted. We also used colors to categorize the type of behavior (gray – positive, red – negative) in the graphs. Therefore, directionality (in terms of who induced a behavior), type of behavior, and strength of interactions were measured. In the graphical representations, the higher thickness of the edges represented higher interactions between the corresponding nodes. In order to make the node strength independent of activities, all edge weights were divided by the largest weight observed for each network to generate normalized weights. Also, to address the varying node sizes, we divided the previously adjusted node strength by the corresponding *n* + 1 values. Thus, global scores were obtained, which were used for the analysis.

*Degree (In and Out-degree)*–Degree of a node was defined by its unique connections to the remaining nodes. In-degree of a node was considered as the number of unique nodes exhibiting any behavior toward it. Similarly, out-degree of a node depicted any behavior originating from it, toward the total number of unique nodes. For example, in a network with six nodes (*N* = 6), node “*i*” can interact with the five (*N* – 1) remaining nodes. Now, if the node “*i*” initiates an interaction or directs behavior toward three unique nodes, it will have an out-degree value of three. On the contrary, if the node “*i*” receives behaviors from four unique nodes, the in-degree value of node “*i*” would be four.

*Degree centrality*–This defines an individual’s structural importance in a network. Degree centrality was calculated by dividing the degree value of a node with the remaining number of nodes in the network. We calculated in and out-degree centrality. Considering the above hypothetical network, “*i*” will have an out-degree centrality [$C_{\text{Out}}^{D}$ (*i*)] value of 0.6 (3/5), and in-degree centrality [$C_{\text{In}}^{D}$ (*i*)] value of 0.8 (4/5).

*Network centrality*–This is the measurement of centrality for an entire network, estimated using the degree centralities of the nodes. We used an index called Network Centrality Index (*NCI^*D*^*) to analyze network centrality (Bhadra et al., 2009). Since we had directed networks, In-degree centrality ($N\,C\,I_{I\,n}^{D}$) and Out-degree centrality ($N\,C\,I_{O\,u\,t}^{D}$) indices were used for better understanding of the data. Network centrality indices were calculated in the following way –

$$N\,C\,I_{I\,n}^{D}=\sum_{i-1}^{n}\left[C_{\text{In}}^{D^{*}}-C_{\text{In}}^{D}\,\left(i\right)\right]/\left(n-1\right)\,\left(n-2\right)$$

[$C_{\text{In}}^{D^{*}}$ = largest observed in-degree value in network N; *n* = Total number of nodes].

Similarly, $N\,C\,I_{O\,u\,t}^{D}$was calculated using the following formula

$$N\,C\,I_{O\,u\,t}^{D}=\sum_{i-1}^{n}\left[C_{\text{Out}}^{D^{*}}-C_{\text{Out}}^{D}\,\left(i\right)\right]/\left(n-1\right)\,\left(n-2\right)$$

[$C_{\text{Out}}^{D^{*}}$ = largest observed out-degree value in network N; *n* = Total number of nodes].

Both the $N\,C\,I_{I\,n}^{D}$and $N\,C\,I_{O\,u\,t}^{D}$values ranged from 0 to 1. A value of 1 indicated a highly centralized network where one of the nodes either initiated all behaviors directed toward others (out-degree) or received all the behaviors from others (in-degree). Therefore these indices provided information on an actor’s (node) role in controlling the network.

*Average clustering co-efficient*–The overall level of clustering or connectedness in a network was measured in addition to network centrality. For example, if a node “*i*” has *k*_*i*_ nodes as neighbors and they are connected, then at most *k*_*i*_(*k*_*i*_−1)/2 edges can exist between them (Watts and Strogatz, 1998). Subsequently, the average value was calculated based on the number of nodes in a network. The values ranged from 0 to 1. A higher clustering coefficient value indicated a more connected network (i.e., stronger interactions among the nodes), whereas a lower value denoted a less connected network (weaker interactions).

Additionally, we calculated the small world (SW) character of the networks. Small-world networks are named with the analogy of “small-world phenomenon” (Milgram, 1967). They are characterized by having higher clustering, and a lower average distance between nodes (Watts and Strogatz, 1998) as compared to random and regular (lattice) networks, respectively. SW was calculated by dividing the clustering coefficient by average distance (Watts and Strogatz, 1998; Bhadra et al., 2009). The range of the SW character was 0–1, with higher values indicating more small-world like networks.

### Statistics

We carried out a generalized linear model (GLM) analysis to understand the effect of human flux zones on the clustering coefficients of the networks, using a Poisson distribution with a “log” link function. A GLM analysis was performed for investigating the effects of human flux zones (Categorical – high/intermediate), and interaction types (Categorical – intraspecific/interspecific) on the number of such interactions. The number of interactions was normalized using the node size of a network. Thus, it allowed us to carry out the analysis across all the networks. We used a Poisson distribution with a “log” link function. We controlled the model for varying network sizes further by adding node size as a control variable.

In the next step, we conducted another GLM analysis to assess the effects of human flux zones and types of *NCI*^*D*^ (Categorical – $N\,C\,I_{I\,n}^{D}$/$N\,C\,I_{O\,u\,t}^{D}$) on the values of *NCI*^*D*^, using a Poisson distribution with a “log” link function. As discussed earlier, *NCI*^*D*^ values were calculated after controlling the different node sizes of the networks. Finally, we investigated the effects of human flux zones (Categorical – high/intermediate), behaviors initiated (Categorical – positive/negative), and initiators (Categorical – dog/human) on the number of interactions using a Poisson distribution with “log” link function. Like earlier, the number of interactions was not absolute values as they were normalized to control for varying node sizes. We also added node size as a control variable in the model.

For all the models, null vs full model comparison was carried out to eliminate Type I error. We first checked the interactive effects of the explanatory variables, in case of no significance, we looked at the individual effects of the predictors. We used the Akaike information criterion (AIC) values (Akaike, 1974) for model selection. We calculated the Δ_*i*_ values by subtracting AIC_*i*_ (AIC of i’th model) from AIC_*min*_ (model with minimum AIC). A Δ_*i*_ of six was followed (Richards, 2005). Residual diagnostics of the models were done using the “DHARMa” package of R (Hartig, 2020). GLM analyses were conducted using “lme4” package of R (Bates et al., 2015). The effect plots were made using the “effects” package of R (Fox and Hong, 2009). The alpha level was 0.05. All statistical analyses were performed using R Studio (version 1.2.5019) (R Development Core Team, 2015). Social network analysis was done using Cytoscape (version 3.8.0) (Shannon, 2003).

## Results

**FIGURE 1:**
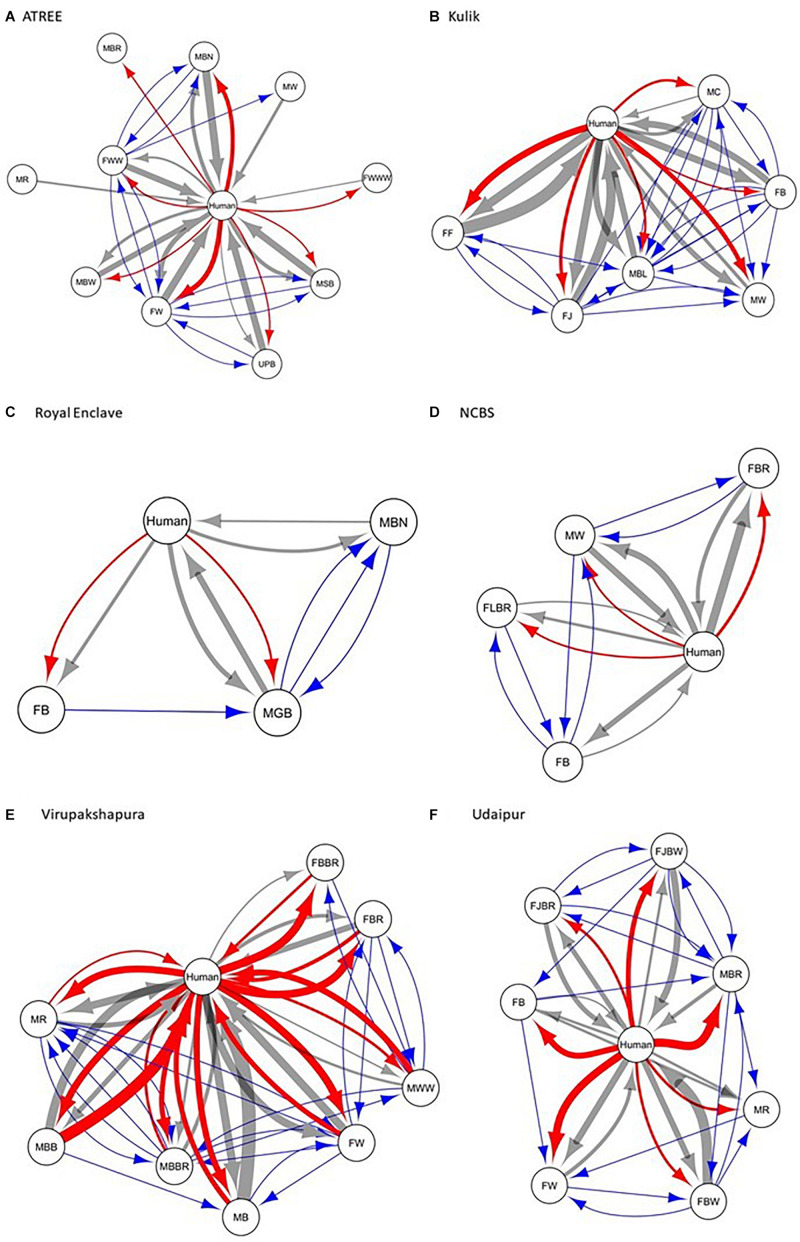
A plot showing the social interactions between dogs and humans in the intermediate human flux zones – **(A)** ATREE, **(B)** Kulik, **(C)** Royal Enclave, **(D)** NCBS, **(E)** Virupakshapura, and **(F)** Udaipur. Circles indicate nodes [dog group members (Supplementary Table S1a) and humans] and connecting lines represent edges. Intra and interspecific interactions are represented by different colors: gray – positive interspecific, red – negative interspecific, blue – intraspecific interactions. All the edges are weighted, indicating the strength of interactions – thicker edges represent stronger interactions, whereas thinner edges represent weaker interactions. All the edges are directed, providing information on nodes initiating and receiving such behaviors.

**FIGURE 2:**
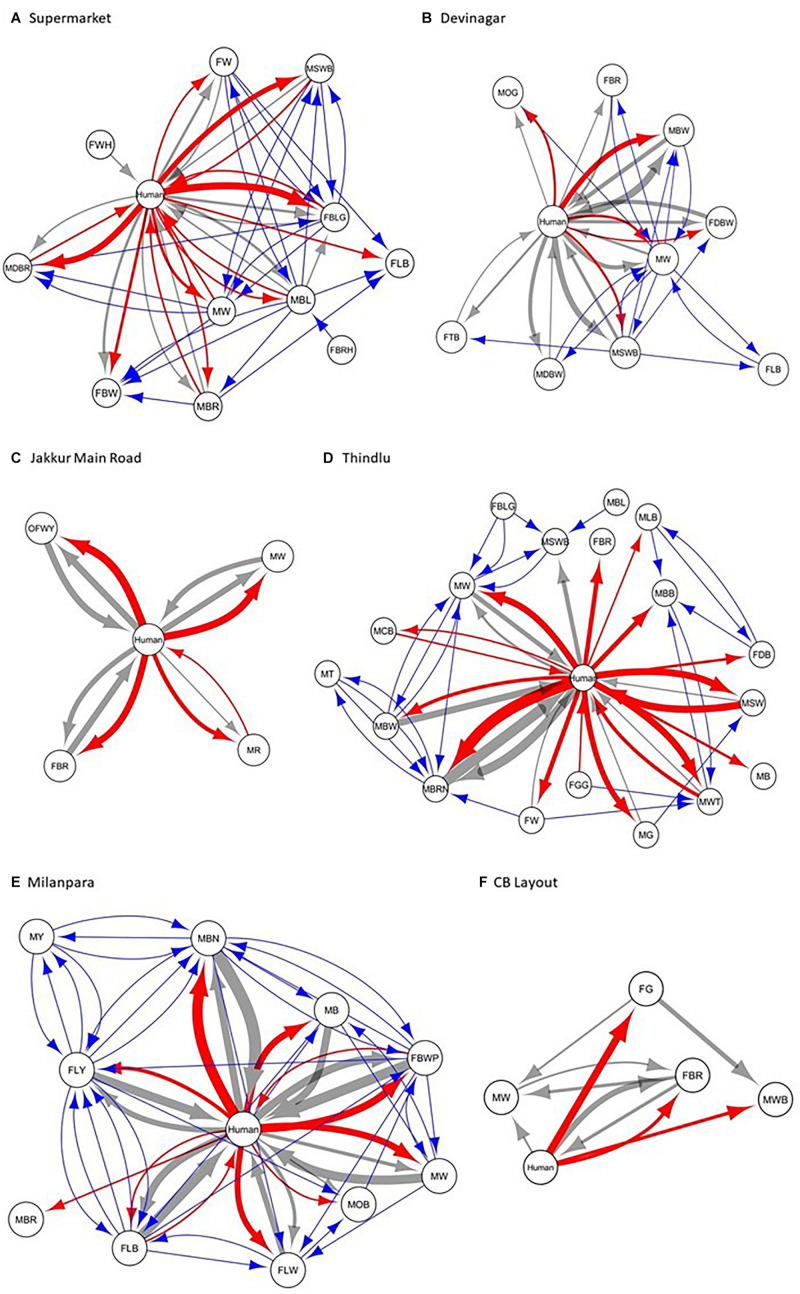
A plot showing the social interactions between dogs and humans in the high human flux zones – **(A)** Supermarket, **(B)** Devinagar, **(C)** Jakkur Main Road, **(D)** Thindlu, **(E)** Milanpara, and **(F)** CB Layout. Circles indicate nodes [dog group members (Supplementary Table S1b) and humans] and connecting lines represent edges. Intra and interspecific interactions are represented by different colors: gray – positive interspecific, red – negative interspecific, blue – intraspecific interactions. All the edges are weighted, indicating the strength of interactions – thicker edges represent stronger interactions, whereas thinner edges represent weaker interactions. All the edges are directed, providing information on nodes initiating and receiving such behaviors.

We constructed the social networks (Figures 1, 2), followed by estimating the network parameters. We summarized the network parameters, including SW and *NCI*^*D*^ in Tables 1, 2 for the intermediate and high human flux zones, respectively.

**TABLE 1:** Table summarizing the network parameters of the intermediate human flux zone networks.

Groups (Networks)	Network parameters						
	*Nodes*	*CL*	*dia*	*d*	*SW*	$N\,C\,I_{I\,n}^{D}$	$N\,C\,I_{O\,u\,t}^{D}$
ATREE	11	0.456	3	1.769	0.26	0.74	0.63
Kulik	6	0.717	2	1.333	0.53	0.46	0.43
Royal Enclave	3	0.75	2	1.333	0.56	0.66	0.66
NCBS	4	0.767	2	1.3	0.58	0.5	0.5
Virupakshapura	8	0.7	2	1.542	0.45	0.71	0.69
Udaipur	7	0.630	3	1.536	0.41	0.42	0.61

**CL*, clustering coefficient; *dia*, network diameter; *d*, average path length; *SW*, small-world value; $N\,C\,I_{I\,n}^{D}$, in-degree network centrality; $N\,C\,I_{O\,u\,t}^{D}$, out-degree network centrality.*

**TABLE 2:** Table summarizing network parameters of the high human flux zone networks.

Groups (Networks)	Network parameters						
	*Nodes*	*CL*	*dia*	*d*	*SW*	$N\,C\,I_{I\,n}^{D}$	$N\,C\,I_{O\,u\,t}^{D}$
Supermarket	12	0.454	3	1.620	0.27	0.54	0.65
Devinagar	7	0.788	3	1.679	0.47	0.55	0.69
Jakkur MR	4	0	2	1.6	0	1	1
Thindlu	15	0.473	5	2.193	0.21	0.51	0.81
Milanpara	10	0.49	3	1.67	0.29	0.38	0.63
CB Layout	4	0.433	3	1.625	0.26	0.5	0.91

**CL*, clustering coefficient; *dia*, network diameter; *d*, average path length; *SW*, small-world value; $N\,C\,I_{I\,n}^{D}$, in-degree network centrality; $N\,C\,I_{O\,u\,t}^{D}$, out-degree network centrality.*

**FIGURE 3:**
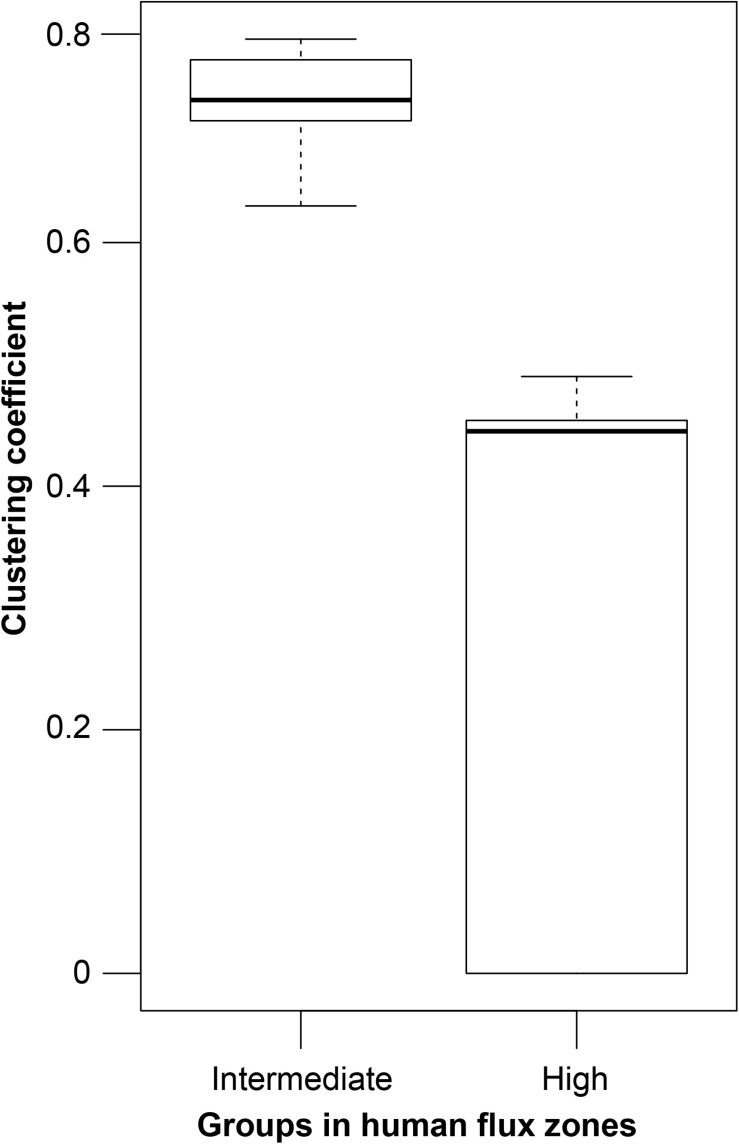
A box and whisker plot showing the network clustering coefficients. Boxes represent the interquartile range, horizontal bars within boxes indicate median values, and whiskers represent the upper range of the data.

GLM analysis revealed significantly higher connectedness of nodes among the networks in the intermediate human flux zones in comparison to the high human flux zones (Table 3 and Figure 3). The average clustering coefficient was found to be 0.67 ± 0.11 and 0.36 ± 0.30 in the intermediate and high human flux zones, respectively. Therefore, the groups were more connected in terms of intra and interspecific interactions in intermediate human flux zones than the high human flux zones.

**TABLE 3:** Generalized linear model showing the effect of human flux zones on the clustering coefficients of the networks.

Fixed effects	Estimate	Std. error	*z*-value	Pr(>| *z*|)
Intercept	3.58352	0.06804	52.667	<2e-16***
Human flux “intermediate”	0.61619	0.08444	7.298	2.93e-13***

***p = 0.

**FIGURE 4:**
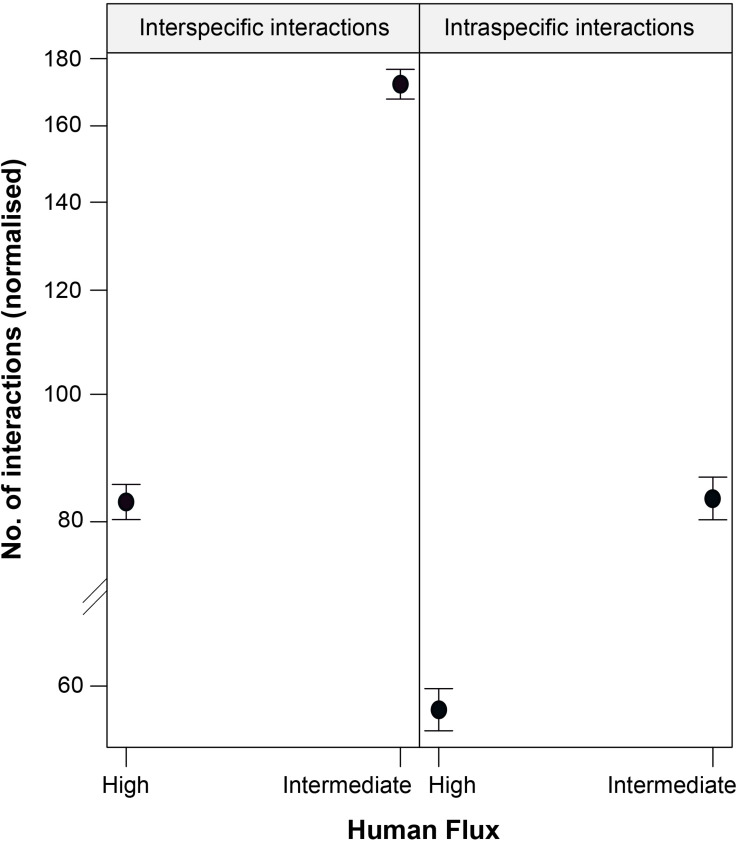
An effect plot showing the interactive effect of human flux (high and intermediate) and type of interactions (intraspecific and interspecific), predicting the number of instances of such interactions. Solid circles and whiskers indicate the mean values and standard errors, respectively.

We found a significant interaction effect between the two kinds of zones and interaction types predicting the instances of such interactions (Table 4 and Figure 4). Interestingly, we noticed significantly higher instances of interspecific interactions in the intermediate human flux as compared to the high flux zones (*p* < 0.001). Intraspecific interactions were also found to be significantly higher in the intermediate than in the high human flux zones (*p* < 0.001). Therefore, dogs were actively interacting with conspecifics and with humans more in the intermediate as compared to the high human flux zones.

**TABLE 4:** Generalized linear model showing the interactive effects of human flux zones and interaction types on the number of such interactions.

Fixed effects	Estimate	Std. error	*z*-value	Pr(>| *z*|)
Intercept	5.406298	0.031181	173.38	<2e-16***
Human flux “intermediate”	0.441334	0.02203	20.03	<2e-16***
Type “intraspecific”	−0.363599	0.024513	−14.83	<2e-16***
Intermediate * intraspecific	−0.361752	0.033772	−10.71	<2e-16***

***p = 0.

**FIGURE 5:**
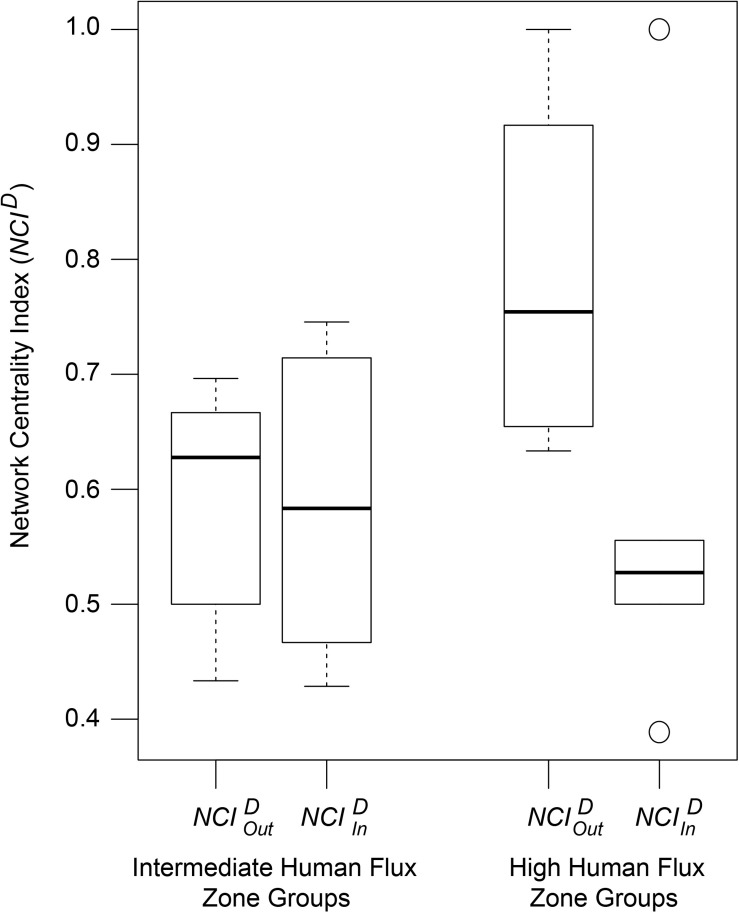
A box and whisker plot showing the Network Centrality Index (*NCI*^*D*^) values of the social networks. Boxes represent the interquartile range, horizontal bars within boxes indicate median values, and whiskers represent the upper range of the data.

*NCI^*D*^* values of the networks were found to be predicted by an interactive effect of human flux zones and *NCI*^*D*^ types (Table 5 and Figure 5). $N\,C\,I_{O\,u\,t}^{D}$was found to be significantly higher in the high human flux zone than the intermediate zone networks, suggesting higher centrality in terms of initiation of behaviors by one of the nodes. Further investigation revealed that the node ‘human’ was responsible for initiating behaviors ($N\,C\,I_{O\,u\,t}^{D}$ = 0.78 ± 0.15) toward dogs, therefore causing increased centrality in the networks.

**TABLE 5:** Generalized linear model showing the interactive effects of human flux zones and types of *NCI*^*D*^ on the values of *NCI*^*D*^.

Fixed effects	Estimate	Std. error	*z*-value	Pr(>| *z*|)
Intercept	4.057565	0.053683	75.584	<2e-16***
*NCI*^*D*^ type “$N\,C\,I_{O\,u\,t}^{D}$”	0.301278	0.070810	4.255	2.09e-05***
Human flux “intermediate”	0.008608	0.07575	0.114	0.90953
$N\,C\,I_{O\,u\,t}^{D}$ * intermediate	−0.29558	0.103499	−2.856	0.00429**

**p = 0.001, ***p = 0.

**FIGURE 6:**
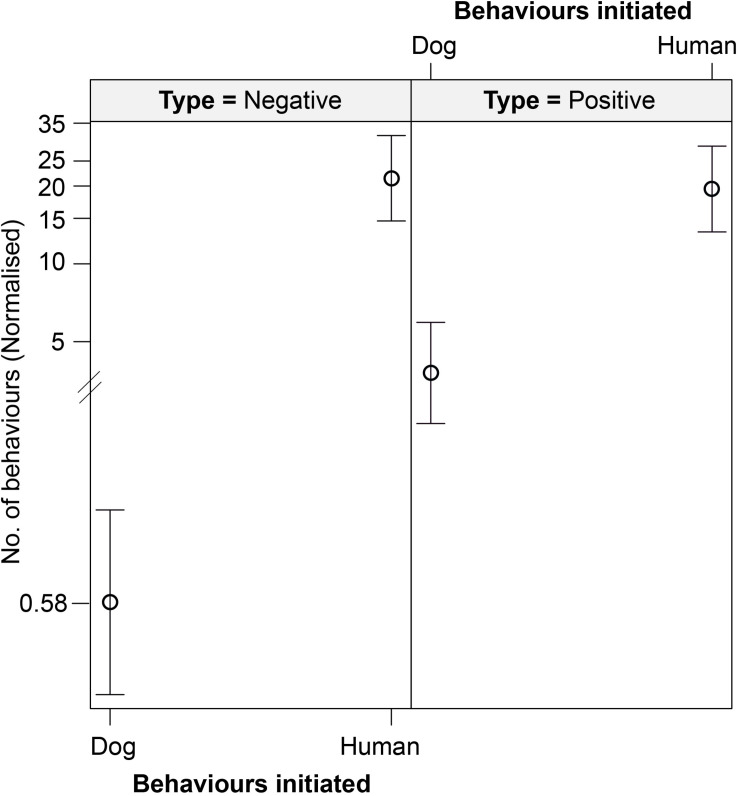
An effect plot showing the interactive effect of type of behavior initiated (positive and negative) and initiator (dog and human), predicting the number of interspecific interactions. Solid circles and whiskers indicate the mean values and standard errors, respectively.

We found an interactive effect between the type of behavior initiated and initiator, predicting the number of interspecific interactions (Table 6 and Figure 6). Humans were found to initiate both positive (*p* < 0.001) and negative (*p* < 0.001) behaviors in significantly higher instances than the dogs. We did not see any impact of human flux zones.

**TABLE 6:** Generalized linear model showing the interactive effects of initiator, and the type of behavior initiated, on the number of interactions.

Fixed effects	Estimate	Std. error	*z*-value	Pr(>| *z*|)
Intercept	−1.15787	0.39591	−2.925	0.00345**
Initiator “Human”	3.77767	0.38226	9.882	<2e-16***
Type of behavior “Positive”	2.04307	0.40172	5.086	3.66e-07***
Zone “Intermediate”	0.10053	0.08517	1.180	0.23785
“Human” * “Positive”	−2.13904	0.41017	−5.215	1.84e-07***

**p = 0.001, ***p = 0.

## Discussion

Our findings clearly suggest a central role of humans in the social interaction networks of free-ranging dogs in India. Contrary to our prediction, interspecific interactions were higher in the intermediate than in the high human flux zones. This indicates that human flux alone cannot be predictive of the magnitude of interspecific interactions. It was further corroborated by the higher average network clustering coefficients in the intermediate human flux zones, pointing to higher connectivity within these networks than in the high human flux zones. Dog-initiated behaviors toward humans were, overall, more positive than negative. Additionally, $N\,C\,I_{O\,u\,t}^{D}$was close to 0.8 in the high flux zones due to significantly higher levels of human-initiated behavior toward dogs. Humans initiated both positive and negative behaviors comparatively more than the dogs. In other words, humans played a vital role in the dynamics of social interaction networks of these dogs.

Previous studies investigating dog–human interactions have suggested pet dogs’ inclination toward preferring a human partner over conspecifics (Kaminski and Marshall-Pescini, 2014; Bräuer, 2015; Nagasawa et al., 2015). However, in free-ranging dogs, intraspecific interactions are necessary for maintaining group stability, defending territories, and other social behaviors, for example, parental care (Pal et al., 1998; Pal, 2003; Bonanni and Cafazzo, 2014; Paul et al., 2014a, b, 2015, 2017). Additionally, a substantial amount of negative human impact has also been suggested (Paul et al., 2016). Similar to other species living in urban habitats, a general aversion toward humans was thus expected (Raussi, 2003; Rodewald and Shustack, 2008; Carrete and Tella, 2011). Hence, it was surprising to observe higher interspecific than intraspecific interactions in these dogs. A recent study also concluded that domestication had shaped free-ranging dogs’ behavior in terms of their tendencies to be in proximity to humans despite their limited socialization experience with humans than pets (Lazzaroni et al., 2020). We speculate that the interactions among conspecifics of a free-ranging dog group may be maintained using subtle behavioral cues. Thus, in a way, they might prefer being in the closeness of conspecifics without showing much direct behavioral interactions. Exploratory studies would be required to understand the presence of such subtle cues (if any) and the underlying dynamics better.

Dog–human relationships have been shown to vary within and across social contexts (Serpell, 2016). Though human flux could not predict the dog and human-initiated positive and negative behaviors toward each other, humans were indeed found to be controlling the network dynamics in the high flux zones. Moreover, investigating the behavior of a species that interacts with humans could be useful to predict the perception or the influence of humans on that species. The differential results of the network properties in the two human flux zones, thus, may be attributed to varying anthropogenic impact on free-ranging dogs. For example, it is known that a personality trait like sociability (Sloan Wilson et al., 1994; Tuomainen and Candolin, 2011), is likely to be shaped by differential human actions through variable ontogenic experiences. As mentioned earlier, free-ranging dogs differ in their sociability behavior with regard to varying human flux (Bhattacharjee et al., 2020a); in crowded areas, dogs are typically exposed to a lot of unfamiliar humans which may eventually facilitate opportunistic begging, while also exposing the dogs to more frequent threats and aggression in other forms from humans. On the contrary, intermediate human flux zones represent areas where dogs encounter less number of unfamiliar humans. It is also important to note that the high human flux zones allow significantly higher access to potential food resources for these dogs than the intermediate ones (Bhattacharjee and Bhadra, under prep.). Unraveling the various factors concerning dog–human interactions will require future studies.

One potential shortcoming of the study was a restricted approach of analysis based on the “initiated” behaviors. We deliberately used the method to have initial baseline information on the dog and human-initiated behaviors toward each other. Further assessment of two-way interactions could be useful to complete the picture in future with more observational studies in specific directions. This is a first attempt to quantify direct interactions between dogs and humans on the Indian streets, providing significant inputs on the scantily explored topic of the free-ranging dog–human relationship. In India, dog–human conflict is a burgeoning issue (Kumar and Paliwal, 2015; Home et al., 2017) which attracts very harsh reactions, and immediate steps are required to curb this. While the law permits animal birth control as the solution to the growing dog population and the mitigation of conflict, this has not yet proven to be a feasible option in a country as large as India. Hence, efficient management of free-ranging dog populations requires a good understanding of their behavior, especially their interactions with humans. Findings from our study may be beneficial in terms of designing better management strategies and mitigation measures for such conflict.

## Data Availability Statement

The raw data supporting the conclusions of this article will be made available by the authors, without undue reservation.

## Ethics Statement

The animal study was reviewed and approved by IISER Kolkata Animal Ethics Committee (approval no. 1385/ac/10/CPCSEA).

## Author Contributions

DB and AB designed and conceived the study. DB carried out the fieldwork and analyzed the data. DB wrote the first draft of the manuscript. AB edited the manuscript and supervised the entire work. Both authors contributed to the article and approved the submitted version.

## Conflict of Interest

The authors declare that the research was conducted in the absence of any commercial or financial relationships that could be construed as a potential conflict of interest.
