# Supplementary material for: Humans Dominate the Social Interaction Networks of Urban Free-Ranging Dogs in India
Source: Front Psychol. 2020 Aug 25;11:2153. doi: 10.3389/fpsyg.2020.02153 (PMC7477117; doi:10.3389/fpsyg.2020.02153)
Supplement: Supplementary file 1 [file Data_Sheet_1.PDF]

Supplementary Information

Humans dominate the social interaction networks of urban free-ranging dogs in India

Debottam Bhattacharjee[1] and Anindita Bhadra[1*]

Affiliation:

[1] Department of Biological Sciences, Indian Institute of Science Education and Research Kolkata, Nadia, West Bengal, India.

[*]**Address for Correspondence**
Behaviour and Ecology Lab, Department of Biological Sciences,
Indian Institute of Science Education and Research Kolkata
Mohanpur Campus, Mohanpur, Nadia
PIN 741246, West Bengal, India.

[*]Corresponding author
E-mail: abhadra@iiserkol.ac.in (AB)

tel. +91-33 6136 0000 ext 1223
fax +91-33-25873020

Table S1 – Table showing the details of the groups observed in the (a) intermediate human flux zones, and (b) high human flux zones.

(a)

Name	Duration	Location	Gr. size	Age class	Sex	Identity
ATREE	Jun – Jul’18	Bengaluru	11	A	M	MW
				A	M	MR
				A	M	MBN
				A	M	MBW
				A	M	MSB
				A	M	MBR
				A	F	FW
				A	F	FWW
				A	F	FWWW
				A	F	FSW
				J	U	UPB
Kulik	Aug – Sept’18	Raiganj	6	A	M	MW
				A	M	MC
				A	M	MBL
				A	F	FB
				A	F	FF
				J	F	FJ
Royal Enclave	Mar – Apr’19	Bengaluru	3	A	M	MGB
				A	F	FB
				A	M	MBN
NCBS	June’19	Bengaluru	4	A	M	MW
				A	F	FB
				A	F	FBR
				A	F	FLBR
Virupakshapura	Jun – Jul’18	Bengaluru	8	A	M	MB
				A	M	MBB
				A	M	MBBR
				A	M	MR
				A	M	MWW
				A	F	FW
				A	F	FBR
				A	F	FBBR
Udaipur	Oct - Nov’18	Raiganj	7	A	M	MBR
				A	M	MR
				A	F	FB
				A	F	FW
				A	F	FBW
				J	F	FJBW
				J	F	FJBR

(b)

Name	Duration	Location	Gr. size	Age class	Sex	Identity
Supermarket	Oct – Nov'18	Raiganj	12	A	F	FLB
				A	F	FWH
				A	F	FBW
				A	F	FBLG
				A	F	FW
				A	F	FBRH
				A	M	MBL
				A	M	MDBR
				A	M	MW
				A	M	MOB
				A	M	MBR
				A	M	MSWB
Devinagar	Nov – Dec'18	Raiganj	7	A	M	MOG
				A	M	MDBW
				A	M	MW
				A	M	MBW
				A	M	MSWB
				A	F	FDBW
				A	F	FBR
Jakkur Main	Feb – Mar'19	Bengaluru	4	A	M	MW
				A	M	MR
				A	F	FBR
				A	F	OFWY
Thindlu	July'18	Bengaluru	15	A	M	MBRN
				A	M	MW
				A	M	MSW
				A	M	MBW
				A	M	MCW
				A	M	MWT
				A	M	MB
				A	M	MBB
				A	M	MG
				A	M	MT
				A	M	MLB
				A	F	FW
				A	F	FDB
				A	F	FGG
				A	F	FBR
Milanpara	Nov – Dec'18	Raiganj	10	A	M	MBN
				A	M	MW
				A	M	MOB
				A	M	MB
				A	M	MBR
				A	M	MY
				A	F	FLB
				A	F	FLW

				A	F	FLY
				A	F	FBWP
CB Layout	Aug’19	Bengaluru	4	A	M	MW
				A	M	MWB
				A	F	FBR
				A	F	FG

Figure S1 – Map showing the study locations. (a) Two cities are pointed in an Indian map where the study was carried out (Raiganj, West Bengal and Bengaluru, Karnataka**) (b) Groups observed in Raiganj, West Bengal**, **(c) Groups observed in Bengaluru, Karnataka.** Green circles indicate intermediate human flux zone groups and red circles indicate high human flux zone groups.

(a)

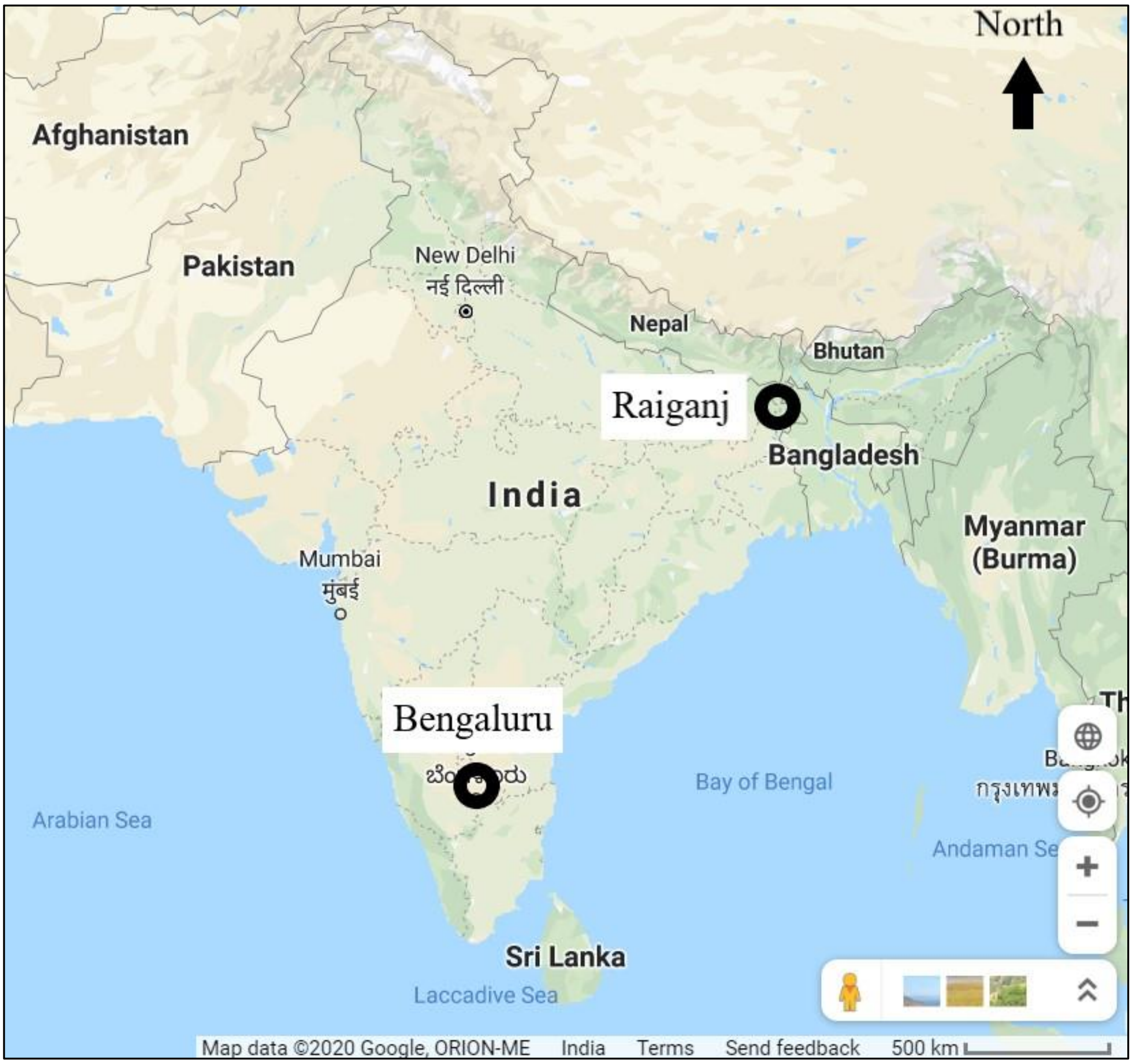

(b)

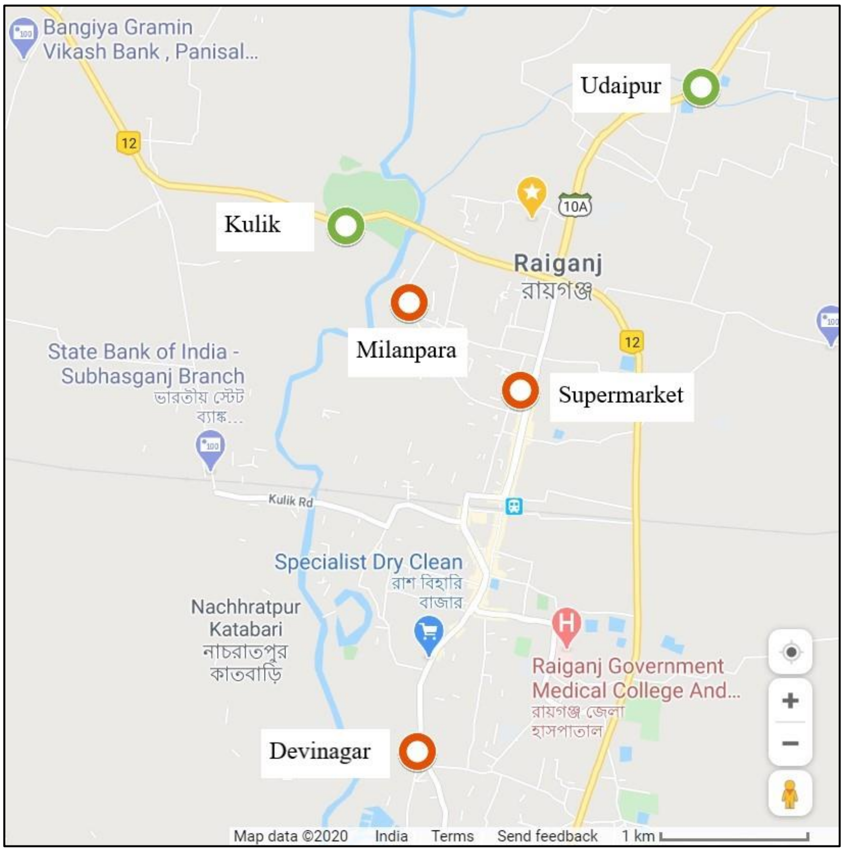
Bangiya Gramin
Vikash Bank , Panisal...
Udaipur
12
10A
Kulik
Raiganj
রায়গঞ্জ
Milanpara
12
State Bank of India -
Subhasganj Branch
Supermarket
Kulik Rd
Specialist Dry Clean
Nachhratpur
Katabari
Raiganj Government
Medical College And...
Devinagar
Map data ©2020 India Terms Send feedback 1 km

(c)

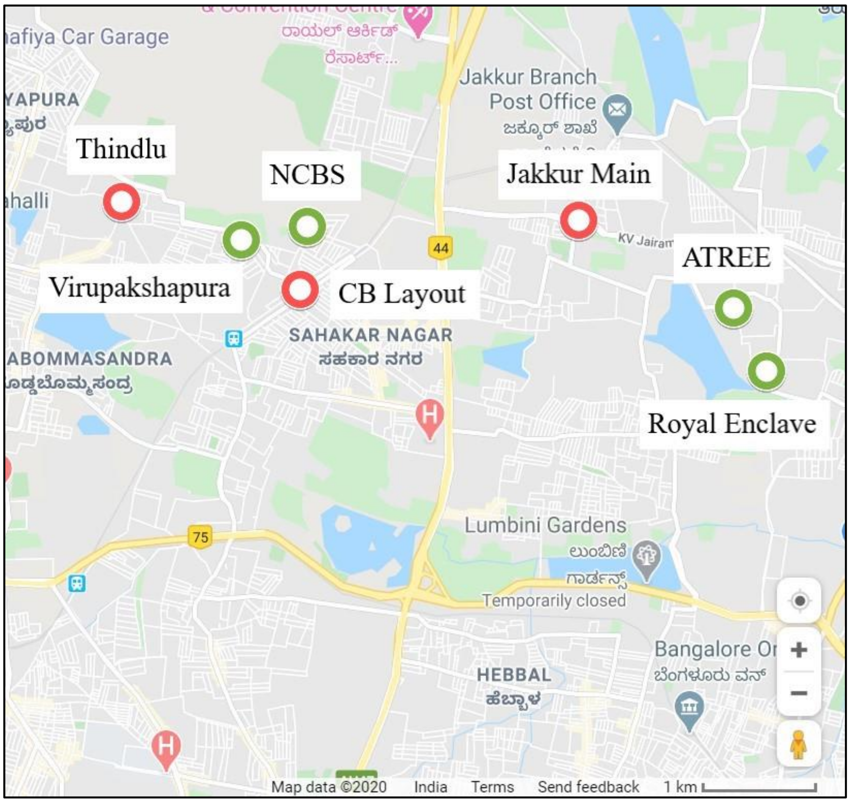
afiya Car Garage
Jakkur Branch
Post Office
Thindlu
NCBS
Jakkur Main
44
Virupakshapura
CB Layout
ATREE
SAHAKAR NAGAR
Royal Enclave
75
Lumbini Gardens
Temporarily closed
HEBBAL
Map data ©2020 India Terms Send feedback 1 km
